# URB937 Prevents the Development of Mechanical Allodynia in Male Rats with Trigeminal Neuralgia

**DOI:** 10.3390/ph16111626

**Published:** 2023-11-18

**Authors:** Chiara Demartini, Rosaria Greco, Anna Maria Zanaboni, Miriam Francavilla, Sara Facchetti, Cristina Tassorelli

**Affiliations:** 1 Department of Brain and Behavioral Sciences, University of Pavia, Via Bassi 21, 27100 Pavia, Italy; annamaria.zanaboni@unipv.it (A.M.Z.); miriam.francavilla@mondino.it (M.F.); cristina.tassorelli@unipv.it (C.T.); 2 Section of Translational Neurovascular Research, IRCCS Mondino Foundation, Via Mondino 2, 27100 Pavia, Italy; rosaria.greco@mondino.it (R.G.); sara.facchetti@mondino.it (S.F.)

**Keywords:** trigeminal neuralgia, FAAH inhibitor, pain, inflammation

## Abstract

Cannabinoids are proposed for alleviating neuropathic pain, but their use is limited by cannabimimetic side effects. The inhibition of the fatty acid amide hydrolase (FAAH), the degrading enzyme of the endocannabinoid anandamide, has received attention as an alternative to cannabinoids in the treatment of neuropathic pain. Here, we investigated the effect of URB937, a blood–brain barrier impermeant FAAH inhibitor, on experimentally induced mechanical allodynia in an animal model of trigeminal neuralgia. Male Sprague-Dawley rats were subjected to chronic constriction injury of the infraorbital nerve (IoN-CCI); operated animals were treated sub-chronically with URB937 (1 mg/kg, i.p.) or vehicle before or after trigeminal mechanical allodynia establishment. We also assayed mRNA expression levels of the pain neuropeptide calcitonin gene-related peptide (CGRP) and cytokines in the medulla, cervical spinal cord, and trigeminal ganglion ipsilateral to IoN-CCI using rt-PCR. URB937 treatment prevented the development of mechanical allodynia and IoN-CCI-induced changes in mRNA expression levels of CGRP and cytokines in the evaluated areas. When administered after allodynia development, URB937 prevented IoN-CCI-induced changes in CGRP and cytokine gene expression; this was not associated with a significant abrogation of the mechanical allodynia. These findings suggest that URB937 may counteract, but not reverse, the development of allodynia in trigeminal neuralgia. Further research is needed to elucidate the underlying mechanisms.

## 1. Introduction

Trigeminal neuralgia is a highly invalidating condition characterized by paroxysmal attacks of sharp pain, frequently described as an electric shock that can severely impact the quality of life of patients. About 50% of affected patients also display persisting pain, which is highly disabling and difficult to treat [1]. The pharmacological treatments available (like anti-convulsants, anti-depressants, and opioids) are often poorly tolerated or ineffective, thus making surgical treatment the only option [2,3,4]. Nonetheless, some patients are refractory to both pharmacological and surgical approaches [5,6]. Therefore, there is a high need to identify new effective and tolerated therapies. 

In recent years, numerous clinical studies and meta-analyses have shown that medicinal cannabis and cannabinoids—by acting on the cannabinoid receptors (CBs) of the endocannabinoid system—are effective in alleviating neuropathic pain [7,8,9,10,11]. Evidence suggests this alternative treatment approach may be suitable also for trigeminal neuralgia [12,13]. Of interest, a case report demonstrated the beneficial effect of a cannabinoid compound in trigeminal neuralgia [14]. The use of cannabinoids in a chronic condition such as trigeminal neuralgia, which usually affects elderly subjects, is limited by their cannabimimetic side effects, like cognitive impairment, dizziness, nausea, and sedation [7,15]. In addition, cannabinoids are associated with a high risk of inducing dependence. In the last decades, the scientific community has put lots of effort into developing alternative approaches that could act on the endocannabinoid system while avoiding the cannabimimetic side effects generated by direct activation of CBs within the CNS [16]. A promising therapeutic tool in this context is represented by the inhibition of the endocannabinoid degrading enzymes, which allows for an enhancement in endocannabinoid tone, possibly lacking the psychotropic effects reported with CB receptor agonists [17,18]. For instance, inhibitors of fatty acid amide hydrolase (FAAH) and monoacylglycerol lipase (MAGL), the enzymes involved in the degradation of the endocannabinoid’s anandamide and 2-arachidonoylglycerol, respectively, were reported to have anti-hyperalgesic and anti-allodynic effects in different models of neuropathic pain [17,18,19]. Most of the pre-clinical studies testing the possible analgesic properties of the endocannabinoid system modulators in neuropathic pain models are those in which the damaged nerves are in extra-cephalic territories (e.g., the sciatic nerve) or in chemotherapy-induced neuropathies [19]. Only a few studies have investigated the effects of endocannabinoid system modulators in neuropathic pain in the orofacial district [17,20,21]. Here, we tested for the first time the effect of a blood–brain barrier (BBB) impermeant FAAH inhibitor [22,23] in an animal model specific for trigeminal neuralgia. Specifically, we aimed to test the ability of the peripherally restricted FAHH inhibitor URB937 in two experimental conditions: (1) prevention/attenuation of the development of trigeminal mechanical allodynia after chronic constriction injury of the infraorbital nerve (IoN-CCI) and (2) reversal of mechanical allodynia induced by IoN-CCI.

## 2. Results

### 2.1. Prevention of Mechanical Allodynia

#### 2.1.1. Mechanical Stimulation Test (MST)

**Figure 1:**
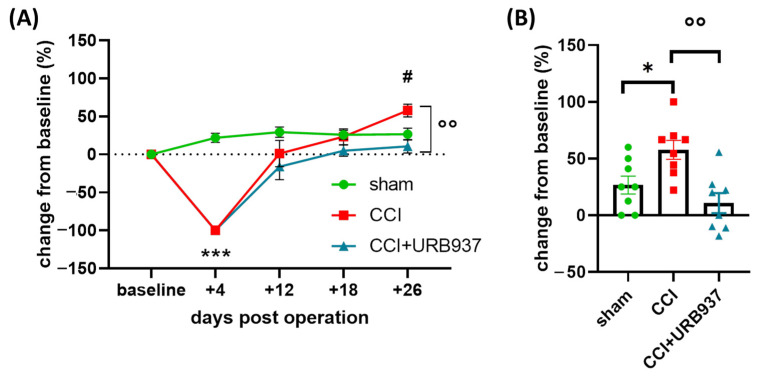
Mechanical stimulation test. (**A**) Change (%) from baseline values to Von Frey hair stimulation (ipsilateral side) on the pre-operative day (baseline) and +4, +12, +18, and +26 days post-operation. Data are expressed as mean ± SEM. Two-way ANOVA followed by Tukey’s multiple comparison test. *** *p* < 0.001 CCI and CCI + URB937 vs. sham; # *p* < 0.05 CCI vs. sham; °° *p* < 0.01 CCI vs. CCI + URB937. (**B**) Change (%) from baseline values to Von Frey hair stimulation (ipsilateral side) on day +26 post-surgery. Data are expressed as mean ± SEM. One-way ANOVA followed by Tukey’s multiple comparison test. * *p* < 0.05 CCI vs. sham and °° *p* < 0.01 CCI vs. CCI*URB937. N = 8.

In accordance with previous studies [24,25], after an initial hyporesponsiveness (day +4), the IoN-CCI-operated animals showed a progressive increase in the mechanical response score ipsilaterally to the surgery (Figure 1A). On day +26, the operated rats (CCI group) displayed a significant increase in the mechanical stimulation test (MST) response (on the side ipsilateral to the operation) compared with the sham-operated animals (Figure 1). Of note, the sub-chronic treatment with URB937 in the operated animals (CCI + URB937 group) significantly attenuated the development of mechanical allodynia (Figure 1), thus supporting the rationale for using FAAH inhibition in the prevention of trigeminal mechanical allodynia.

#### 2.1.2. Gene Expression

**Figure 2:**
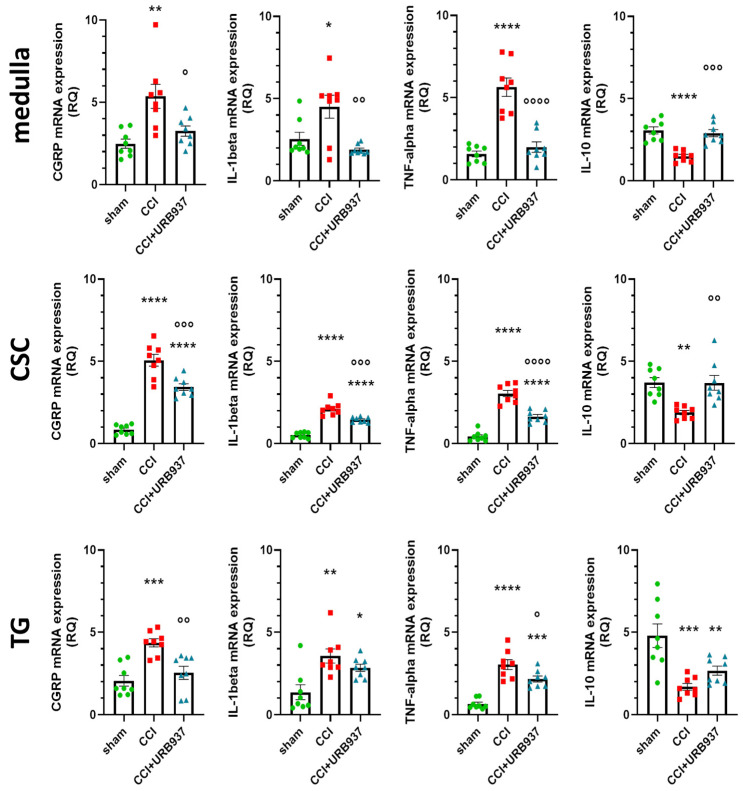
Gene expression levels (expressed as relative quantification, RQ) of CGRP, IL-1beta, and TNF-alpha e IL-10 in the medulla, cervical spinal cord (CSC), and trigeminal ganglion (TG) ipsilateral to the IoN-CCI (day +26). Data are expressed as mean ± SEM. One-way ANOVA followed by Tukey’s multiple comparison test. * *p* < 0.05, ** *p* < 0.01, *** *p* < 0.001 and **** *p* < 0.0001 vs. sham; ° *p* < 0.05, °° *p* < 0.01, °°° *p* < 0.001 and °°°° *p* < 0.0001 vs. CCI. N = 8.

In the cervical spinal cord (CSC) and trigeminal ganglion (TG) ipsilateral to the IoN-CCI and in the medulla, the mRNA expression levels of calcitonin gene-related peptide (CGRP), interleukin (IL)-1beta, and tumour necrosis factor-alpha (TNF-alpha) were significantly higher in the CCI group compared with the sham group, confirming previous results [24]. Such increases were significantly attenuated by the URB937 sub-chronic treatment (CCI + URB937 group) except for IL-1beta in the TG area. Regarding the anti-inflammatory cytokine IL-10, the operated animals (CCI group) showed reduced gene expression levels compared with the sham group, and the URB937 treatments restored the IL-10 gene expression levels in the medulla and CSC, but not in the TG. These data, shown in Figure 2, suggest that in the prevention of mechanical allodynia, URB937 acts by the modulation of nociceptive and inflammatory pathways in specific areas linked to trigeminal pain transmission.

### 2.2. Reversal of Mechanical Allodynia

#### 2.2.1. Mechanical Stimulation Test (MST)

**Figure 3:**
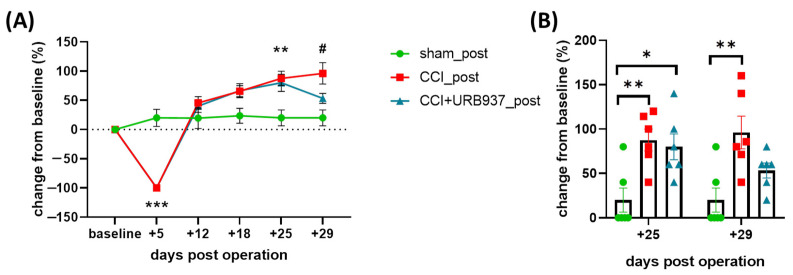
Effect of URB937 on the mechanical stimulation test response in allodynic rats. URB937 was administered from day +25 to +29 post-surgery (5 total injections). (**A**) Change (%) from baseline values to Von Frey hair stimulation (ipsilateral side) on the pre-operative day (baseline) and +5, +12, +18, +25, and +29 days post-operation. Data are expressed as mean ± SEM. Two-way ANOVA followed by Tukey’s multiple comparisons test; ** *p* < 0.01 and *** *p* < 0.001 CCI_post and CCI + URB937_post vs. sham_post; # *p* < 0.05 CCI_post vs. sham_post. (**B**) Change (%) from baseline values to Von Frey hair stimulation (ipsilateral side) on day +25 and +29 post-surgery. Data are expressed as mean ± SEM. One-way ANOVA followed by Tukey’s multiple comparison test. ** *p* < 0.01 CCI_post vs. sham_post; * *p* < 0.05 CCI + URB937_post vs. sham_post. N = 6.

Comparable to previous data, the initial hyporesponsiveness in the MST (day +5) displayed by the IoN-CCI-operated animals was gradually replaced by hyperresponsiveness ipsilaterally to the surgery (Figure 3A). Indeed, on day +25, the IoN-CCI-operated animals (both the CCI_post and CCI + URB937_post groups) showed a significant increase in the MST response (on the side ipsilateral to the operation) compared with the sham-operated animals (Figure 3). The treatment with URB937 after the complete development of mechanical allodynia was not able to significantly reverse it, although a tendency for a reduction was appreciable (CCI + URB937_post group on day +29) (Figure 3). This finding indicates that URB937 is unable to reverse trigeminal mechanical allodynia once it has developed.

#### 2.2.2. Gene Expression

**Figure 4:**
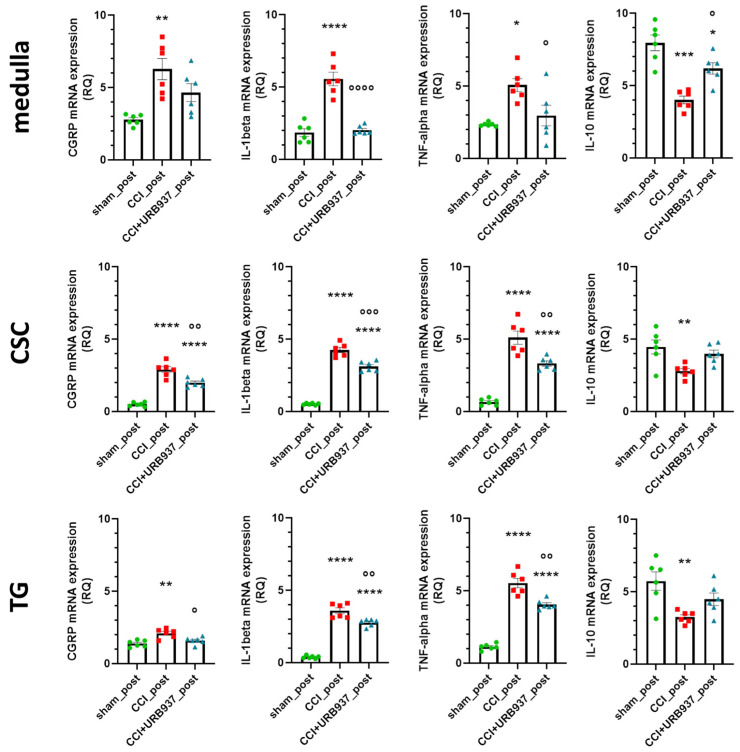
Gene expression levels (expressed as relative quantification, RQ) of CGRP, IL-1beta, and TNF-alpha e IL-10 in the medulla, cervical spinal cord (CSC), and trigeminal ganglion (TG) ipsilateral to the IoN-CCI (day +29 post-surgery, after 5 daily URB937 injections). Data are expressed as mean ± SEM. One-way ANOVA followed by Tukey’s multiple comparison test. * *p* < 0.05, ** *p* < 0.01, *** *p* < 0.001 and **** *p* < 0.0001 vs. sham_post; ° *p* < 0.05, °° *p* < 0.01, °°° *p* < 0.001, and °°°° *p* < 0.0001 vs. CCI_post. N = 6.

In the medulla, CSC, and TG ipsilateral to the IoN-CCI, the mRNA expression levels of CGRP, IL-1beta, and TNF-alpha were significantly higher in the CCI_post group compared with the sham_post group, confirming previous results [24]. Such an increase was significantly reduced with the URB937 chronic treatment (CCI + URB937_post group), except for CGRP in the medulla area. Regarding the anti-inflammatory cytokine IL-10, the operated animals (CCI_post group) showed reduced gene expression levels compared with the sham_post group; URB937 chronic treatments changed the IL-10 gene expression levels only in the medulla area. These data, shown in Figure 4, suggest that URB937 can modulate mediators of inflammation, but it does not reverse MST. 

## 3. Discussion

To date, there is considerable evidence supporting the effectiveness of cannabinoids and modulators of the endocannabinoid system in alleviating experimentally induced neuropathic pain [17,18], including in models specific to the trigeminal district [20,21]. The use of inhibitors of endocannabinoid-degrading enzymes allows for the maintenance of endocannabinoid tone, and consequently, the activation of CB receptors only in the body sites where they are needed [26], thus limiting unwanted central side effects. 

Here, we tested for the first time the BBB impermeant FAAH inhibitor URB937 [22,23] in the pre-clinical model of trigeminal neuralgia based on IoN-CCI. Our data confirm the anti-allodynic effect of URB937, as previously reported in other neuropathic pain models, namely, sciatic nerve CCI [22,27,28], chemotherapy-induced neuropathy [29,30,31], and diabetic peripheral neuropathy [32]. In this study, we found that sub-chronic treatment with URB937 was able to prevent the development of trigeminal mechanical allodynia but not to reverse it once developed, although a pattern towards reduction was visible. It is possible that the lack of significant efficacy in allodynia reversal may be due to intergroup variability; alternatively, the URB937 treatments scheduled for the abrogative set (five total injections) were not sufficient to significantly counteract the already-established allodynia. Nonetheless, in both experimental paradigms, the FAAH inhibitor proved to be able to reduce the IoN-CCI-induced upregulation of the pain neuropeptide CGRP and pro-inflammatory cytokines TNF-alpha and IL-1beta and to increase the anti-inflammatory cytokine IL-10 in trigeminal-related areas. The present findings are consistent with previous studies in which FAAH inhibitors were able to alter the gene expression of pain and inflammatory mediators [33,34,35,36,37,38]. However, based on the discrepancies observed in these two different experimental settings, it appears that changes in gene expression of the mediators evaluated in this study are not directly associated with mechanical pain, thus suggesting that other signalling pathways and mediators are involved. 

Here, we found molecular changes induced with URB937, not only at the peripheral level (i.e., TG) but also centrally (i.e., medulla and CSC) where the trigeminal nucleus caudalis (receiving trigeminal afferents) is located. It is true that URB937 is a peripherally restricted FAAH inhibitor [22,23], but it is possible that the effect observed in the medulla and CSC regions could be related to the penetrance of URB937 through the area postrema, a brainstem region that is not protected by the BBB. Accordingly, in the area postrema, Rock and collaborators [39] found reduced FAAH activity after URB937 treatment, together with increased levels of anandamide, oleoylethanolamide, and palmitoylethanolamide (PEA). Furthermore, in an animal model of migraine, we previously reported an increase in PEA levels in the medulla area after URB937 treatment, associated with changes in cytokines gene expression [33], partially supporting the central effect of URB937. In addition, it is also likely that a localized [40,41] alteration of the BBB and the blood–spinal cord barrier permeability induced by injury damage [42] could have contributed to URB937 penetrance. Indeed, partial sciatic nerve ligation has been shown to alter the functional and molecular integrity of the blood–spinal cord barrier, restricted to the lumbar spinal cord, through the activation of inflammatory pathways [40]. Thus, we can speculate on the occurrence of a region-specific (trigeminal pathway) BBB disruption following the induction of trigeminal neuropathic pain, which may have allowed URB937 to penetrate to the medulla and CSC level but not to higher cerebral structures, thus avoiding central side effects. Further experiments will be required to assess BBB permeability after induction of trigeminal neuropathic pain and to investigate the putative effects of URB937 on brain areas. 

The molecular and cellular mechanisms through which URB937 induced the changes observed in this study remain to be defined. Nevertheless, URB937 activity is likely mediated by CB1 receptors, as reported in neuropathic pain studies [22,29,31] as well as in another chronic pain model (i.e., migraine) linked with trigeminal pain [33]. Of interest, previous studies reported an upregulation of CB1 receptors in the dorsal root ganglia after spinal nerve ligation [43], in the superficial spinal cord dorsal horn after CCI of the sciatic nerve [44], and also in the superficial laminae of the trigeminal nucleus caudalis after IoN-CCI [21]. This upregulation of CB1 receptors was associated with elevated levels of anandamide in the dorsal root ganglia and spinal cord after the induction of spinal or sciatic nerve ligation [43,45,46]. Elevated anandamide levels were also found in central supraspinal areas implicated in nociception (dorsal raphe, periaqueductal grey and rostral ventral medulla) [45]. CB1 upregulation may be part of the phenotypic and structural reorganization that takes place during the central sensitization process [47]. Central sensitization is characterized by alterations in membrane excitability, reductions in inhibitory transmission, and increases in synaptic efficacy, which converge in the modification of nociceptive circuitry, resulting in an excessive response to noxious (hyperalgesia) or innocuous (allodynia) stimuli [48]. In the dynamic process that leads to the development of allodynia, we can hypothesize that, following FAAH inhibition, a further localized increased tone of anandamide would activate the CB1 receptors in primary afferent neurons of the TG [49,50], causing an indirect effect in central areas. However, chronic FAAH inhibition inhibited the development but not the reversal of allodynia, suggesting that the degree of FAAH inhibition required to reverse allodynia is likely to be greater. It is also possible that URB937 modulates different signalling pathways involved in the initiation and maintenance of pain [51]. For example, activation of c-Jun N-terminal kinase in the dorsal root ganglion and spinal cord plays different roles in regulating the development and maintenance of neuropathic pain induced by spinal nerve ligation [52]. 

In addition, it is possible that the URB937 anti-inflammatory effects are mediated also by anandamide-independent pathways since FAAH can also hydrolyse the endocannabinoid-related lipid PEA, whose levels were found to be increased after URB937-induced FAAH inhibition [22,33]. This lipid amide indeed does not act directly on CB receptors but agonizes other receptors, like peroxisome proliferator-activated receptors (PPARs), thus exerting anti-inflammatory and analgesic effects [53,54,55,56,57]. Specifically, PEA produced and hydrolysed by microglia in response to inflammatory events [58] promotes the expression of anti-inflammatory molecules via PPAR alpha receptors [54]. Therefore, it is possible that penetration of a small amount of URB937 through the BBB at the area postrema level could have inhibited the activity of FAAH expressed by glial cells in the medulla [59,60,61]. 

This is also relevant when considering the importance of neuron–non-neuron interactions and glia–glia cross-talk in the development of peripheral and central sensitization [62,63,64] and in the maintenance of neuropathic pain [63,65,66]. Increased inflammatory mediators sensitize afferent nerves by changing the expression of ion channels, leading to spontaneous pain. Microglia and astrocyte activation lead to overregulation of chemokines and increased signalling between neurons and glial cells. Moreover, neurons can maintain glial cell activation through chemokines and related pathways [67]. Among the different mediators implicated in these interactions, a role is played by cytokines, like IL-1beta [68,69,70], whose expression in the present study was found to be increased in trigeminal areas of operated animals, along with other pro-inflammatory molecules. However, although URB937 modulated the gene expression of inflammatory mediators and CGRP, their expression does not appear to be directly related to trigeminal mechanical allodynia. It is likely that these mediators, together with other factors, are involved in both the development and maintenance of mechanical pain. Since CBs and PPARs are localized also on glial cells [71,72,73], it is likely that at the level of non-neuronal cells, the changes in cytokine expression are directly mediated by endocannabinoids and related lipids, whose anti-inflammatory activity is already known [18,74,75,76,77]. For instance, in neuropathic pain models, the activation of CB receptors reduced the expression of pro-inflammatory agents and increased the anti-inflammatory one [78] probably by the inactivation of transcription factors cAMP response element-binding protein (CREB) and nuclear factor kappa light chain enhancer of activated B cells (NF-κB) [79]. Of interest, anandamide was shown in vitro to inhibit TNF alpha-induced NF-κB activation by direct inhibition of IkappaB kinase [76].

Mounting evidence indeed shows that immune cells can generate and secrete endocannabinoids and may be regulated by them, influencing the production of inflammatory factors [47].

Based on the available literature data, we hypothesize that following nerve injury, several signalling pathways occur at central and peripheral terminals during the development and maintenance of pain. These signalling mechanisms can activate both postsynaptic neurons and glia and increase neuron–glia coupling [66]. In our study, treatment with URB937 inhibited the development of allodynia but not its reversal, probably via an effect on the TG (acting directly on neurons and/or glial cells) that is not yet fully understood. URB937 most likely decreased nociceptive input to the afferent terminals of the trigeminal nerve and to second-order neurons, although a central effect cannot be excluded. However, a direct effect of URB937 at the level of the medulla cannot be ruled out. 

### Limitations of This Study

Despite the encouraging results reported here, this study has some limitations that should be considered in further studies. Firstly, it is necessary to extend the potential efficacy of URB937 in abolishing the mechanical allodynia already established with a different treatment regimen and to evaluate other mediators/pathways that may be involved. Secondly, an assessment of endocannabinoid and related lipid levels in areas of the trigeminal pathway is necessary to confirm the observed efficacy.

Additionally, even though URB937 is a peripherally restricted FAAH inhibitor [22,23] with potentially limited central side effects, the assessment of tolerability, abuse, and side effects assumes great relevance after a sub-chronic/chronic treatment paradigm. However, it should be noted that similar to other FAAH inhibitors that only act where there is increased endocannabinoid release due to an inflammatory state, it is reasonable to assume that URB937 is unlikely to induce central side effects [80,81], such as motor dysfunction [82,83]. In accordance, acute or sub-chronic URB937 treatment showed a tolerability and safety profile [84] and did not induce dependence [31]

Interestingly, within the reversal setting, a subset of the animals underwent the open-field test. After URB937 treatment, no changes in the total distance travelled, average speed, or immobility time were reported (Figure S1). Nonetheless, the putative direct central effects of URB937 following BBB permeability induced with trigeminal nerve injury are worth further investigation.

Finally, another limitation of the present study is the lack of female animals. The influence of sex on pain stimuli is very consistent, with females showing greater sensitivity than males. However, the magnitude of the sex differences varies according to the strain of the animal and the type of study conducted, leading to conflicting results in published studies [85].

## 4. Materials and Methods

### 4.1. Animals and Drugs

A total of 42 adult male Sprague-Dawley rats (weight 200–250 g at arrival) were used. Animals were housed in plastic boxes in groups of two with water and food available ad libitum and kept on a 12:12 h light–dark cycle. All procedures were approved by the Italian Ministry of Health (Document number n° 386/2019-PR) and performed in agreement with the guidelines of the European Community Directive 2010/63/EU of 22 September 2010. Upon arrival, animals were habituated to the housing conditions for 1 week before the experimental testing. 

URB937 (N-cyclohexyl-carbamic acid, 3′-(aminocarbonyl)-6-hydroxy [1,1′-biphenyl]-3-yl ester, Cayman Chemical) was dissolved freshly on treatment days in 10% polyethylene glycol 200, 10% Tween 80, and saline. It was administered intraperitoneally (i.p.) at the dose of 1 mg/kg [33].

### 4.2. Surgery

The IoN-CCI was performed as previously described [24,25]. Rats were anaesthetized with sodium thiopental (50 mg/kg, i.p.). The surgery was performed under direct visual control using a stereo microscope (model PZMTIV-BS, World Precision Instrumens, Sarasota, FL, USA). The rat’s head was fixed in a stereotaxic frame (Stoelting, Ugo Basile, Comerio, Italy), and a mid-line scalp incision was made, exposing the skull and nasal bone. The edge of the orbit was dissected free, and the orbital contents were deflected with a cotton-tipped wooden rod to give access to the left IoN, which was loosely ligated with two chromic catgut ligatures (5-0; Dynec) (2 mm apart). The scalp incision was closed using silk sutures (5-0; Ethicon). In the sham-operated rats, the IoN was exposed using the same procedure, but the nerve was not ligated.

### 4.3. Experimental Groups

#### 4.3.1. Prevention of Mechanical Allodynia

**Figure 5:**
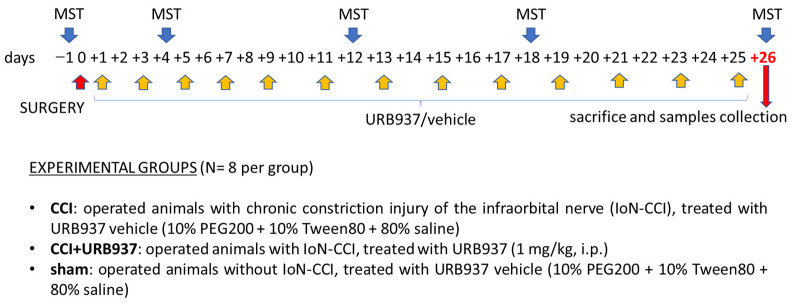
Experimental design of the set for the prevention of mechanical allodynia. Timeline for treatment, mechanical stimulation test (MST) procedures, and experimental groups.

To evaluate the effect of URB937 in preventing the development of trigeminal mechanical allodynia, the IoN-CCI animals were subjected to sub-chronic treatment with URB937 or vehicle. Specifically, the rats were treated with URB937 or vehicle on day +1 after surgery and then every other day until day +25 (Figure 1). The animals were tested with MST (see Section 4.4) the day before surgery (for baseline measurement) and on days +4, +12, +18, and +26 after surgery [24] (Figure 5). On day +26, at the end of MST, the rats were sacrificed, and samples were collected for rt-PCR analysis (see Section 4.5).

#### 4.3.2. Reversal of Mechanical Allodynia

**Figure 6:**
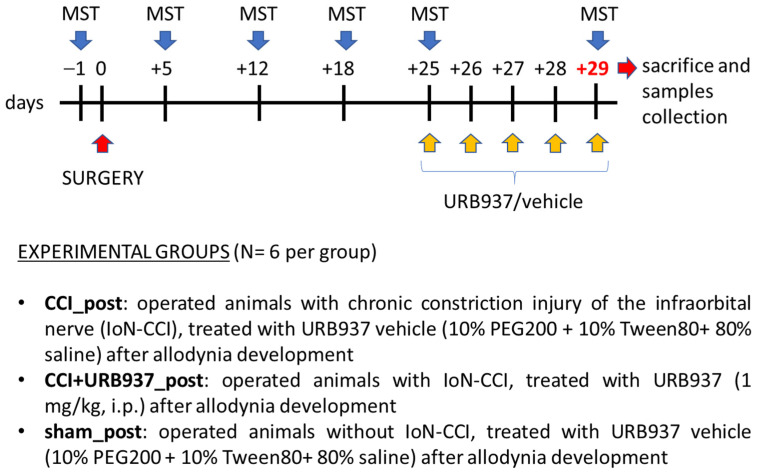
Experimental design of the set for the abrogation of mechanical allodynia. Timeline for treatment, mechanical stimulation test (MST) procedures, and experimental groups.

To evaluate the effect of URB937 in reverting trigeminal mechanical allodynia, a different group of IoN-CCI animals was subjected to chronic treatment with URB937 or vehicle after the complete development of allodynia. The animals underwent the MST (see Section 4.4) the day before surgery (for baseline measurement) and on days +5, +12, +18, and +25 post-surgery. On day +25, after the MST, the rats were treated with URB937 or vehicle daily for 5 days (until day +29) (Figure 6). On day +29, the animals underwent the MST 1 h after URB937 or vehicle treatment; at the end of the MST, the rats were sacrificed, and samples were collected for rt-PCR analysis (see Section 4.5).

### 4.4. Mechanical Stimulation Test (MST)

Baseline data were obtained 1 day before surgery. Following surgery, the rats were tested on post-operative days as described above (Section 4.3., Figure 5 and Figure 6). A graded series of five Von Frey hairs (0.015 g, 0.127 g, 0.217 g, 0.745 g, and 2.150 g) were applied within the IoN territory, near the centre of the vibrissal pad [24,86,87]. The Von Frey hairs were applied in an ascending order of intensity either ipsi- or contralaterally. The scoring system described by Vos [86] was used to evaluate the rats’ response to the stimulation (Table 1). For each rat, and at every designated time, a mean score for the five Von Frey filaments was determined. The final MST responses of each rat are expressed as percentages to its baseline values.

### 4.5. Real Time-PCR

Following the last MST, the rats were sacrificed with a lethal dose of anaesthetic (sodium thiopental, 150 mg/kg, i.p.). To fully include the trigeminal nucleus caudalis [88], the trigeminal nociceptive information including that related to the IoN territory [89,90], the medulla (bregma, −13.30 to −14.60 mm), and the upper CSC (C1-C2 segment) ipsilateral to IoN-CCI was collected, together with the ipsilateral TG. After collection, the samples were put in cryogenic tubes and then immediately frozen in liquid nitrogen and kept at −80 °C until processing.

The mRNA expression levels of the genes coding for the pain neuropeptide CGRP (forward primer: CAGTCTCAGCTCCAAGTCATC; reverse primer: TTCCAAGGTTGACCTCAAAG), the pro-inflammatory cytokines TNF-alpha (forward primer: CCTCACACTCAGATCATCTTCTC; reverse primer: CGCTTGGTGGTTTGCTAC) and IL-1beta (forward primer: CTTCCTTGTGCAAGTGTCTG; reverse primer: CAGGTCATTCTCCTCACTGTC), and the anti-inflammatory cytokine IL-10 (forward primer: GCTCAGCACTGCTATGTTGC; reverse primer: CAGTAGATGCCGGGTGGTTC), were measured using rt-PCR as described elsewhere [24,91]. All samples were assayed in triplicate, and for normalization, the glyceraldehyde 3-phosphate dehydrogenase (GAPDH, forward primer: AACCTGCCAAGTATGATGAC; reverse primer: GGAGTTGCTGTTGAAGTCA) was used. Gene expression levels were calculated using the ΔΔCt method and expressed as relative quantification (RQ).

### 4.6. Statistical Evaluation

The primary outcome of the present study was to evaluate the efficacy of URB937 in preventing/abolishing the development of trigeminal mechanical allodynia generated by IoN-CCI. Based on previous studies that tested URB937 either in a different neuropathic pain model [29] or in other types of chronic pain [33], in CCI + URB937 rats, we hypothesized a response to mechanical stimulation comparable to that of the sham-operated animals. Thus, we considered a difference of at least 40% between CCI and CCI + URB groups and estimated a minimum of six animals per experimental group with an effect size of 0.857. The a priori power analysis (GPower 3.1) was conducted by setting a statistical power of 0.8 at an alpha level of 0.05.

Statistical analyses were performed with the GraphPad Prism program (version 8, GraphPad Software, San Diego, CA, USA). The MST and gene expression data were tested for normality using the Kolmogorov–Smirnov normality test and considered normal. Differences among groups were analysed with one-way or two-way analysis of variance (ANOVA) followed by Tukey’s multiple comparison test depending on the analysis to be performed. A probability level of less than 5% was regarded as significant.

All experiments were conducted by researchers blinded to the treatments.

## 5. Conclusions

The data from this study show that URB937 can abolish but not reverse the development of mechanical allodynia in a model of trigeminal neuropathic pain. This effect does not appear to be mediated by CGRP and inflammatory mediators, confirming that different mechanisms are involved in the induction and maintenance of chronic pain [52]. Thus, further investigations are needed to unravel other mediators and the related mechanisms.

## Figures and Tables

**Table 1:** Response categories with corresponding score values.

Score	Type of Response
0	no response
1	detection: the rat turns its head towards the stimulus object, and the stimulus object is then explored
2	withdrawal reaction: the rat turns its head slowly away or pulls it briskly backwards when the stimulation is applied; sometimes, a single face wipe ipsilateral to the stimulated area occurs
3	escape/attack: the rat avoids further contact with the stimulus object, either passively by moving its body away from the stimulus object to assume a crouching position against the cage wall or actively by attacking the stimulus object, making biting and grabbing movements
4	asymmetric face grooming: the rat displays an uninterrupted series of at least three face-wash strokes directed towards the stimulated facial area

## Data Availability

Data is contained within the article and supplementary material and are openly available in the ZENODO repository (DOI: 10.5281/zenodo.8123831).

## Supplementary Materials

The following supporting information can be downloaded at: https://www.mdpi.com/article/10.3390/ph16111626/s1, Figure S1: Open field test.

## References

[1] Cruccu G. (2017). Trigeminal Neuralgia. Contin. Lifelong Learn. Neurol..

[2] Guirguis-Blake J., Kelly C. (2007). Are Opioids Effective in the Treatment of Neuropathic Pain?. Am. Fam. Physician.

[3] Pergolizzi J.V., Gharibo C., Magnusson P., Breve F., LeQuang J.A., Varrassi G. (2022). Pharmacotherapeutic Management of Trigeminal Neuropathic Pain: An Update. Expert. Opin. Pharmacother..

[4] Al-Quliti K.W. (2015). Update on Neuropathic Pain Treatment for Trigeminal Neuralgia. Neurosciences.

[5] Obermann M., Katsarava Z. (2009). Update on Trigeminal Neuralgia. Expert Rev. Neurother..

[6] Zakrzewska J.M., Akram H. (2011). Neurosurgical Interventions for the Treatment of Classical Trigeminal Neuralgia. Cochrane Database Syst. Rev..

[7] Whiting P.F., Wolff R.F., Deshpande S., Di Nisio M., Duffy S., Hernandez A.V., Keurentjes J.C., Lang S., Misso K., Ryder S. (2015). Cannabinoids for Medical Use. JAMA.

[8] Wallace M.S., Marcotte T.D., Umlauf A., Gouaux B., Atkinson J.H. (2015). Efficacy of Inhaled Cannabis on Painful Diabetic Neuropathy. J. Pain.

[9] Wilsey B., Marcotte T., Deutsch R., Gouaux B., Sakai S., Donaghe H. (2013). Low-Dose Vaporized Cannabis Significantly Improves Neuropathic Pain. J. Pain.

[10] Serpell M., Ratcliffe S., Hovorka J., Schofield M., Taylor L., Lauder H., Ehler E. (2014). A Double-Blind, Randomized, Placebo-Controlled, Parallel Group Study of THC/CBD Spray in Peripheral Neuropathic Pain Treatment. Eur. J. Pain.

[11] Sainsbury B., Bloxham J., Pour M.H., Padilla M., Enciso R. (2021). Efficacy of Cannabis-Based Medications Compared to Placebo for the Treatment of Chronic Neuropathic Pain: A Systematic Review with Meta-Analysis. J. Dent. Anesth. Pain Med..

[12] Liang Y.C., Huang C.C., Hsu K.S. (2004). Therapeutic Potential of Cannabinoids in Trigeminal Neuralgia. Curr. Drug Target CNS Neurol. Disord..

[13] McDonough P., McKenna J.P., McCreary C., Downer E.J. (2014). Neuropathic Orofacial Pain: Cannabinoids as a Therapeutic Avenue. Int. J. Biochem. Cell Biol..

[14] Gajofatto A. (2016). Refractory Trigeminal Neuralgia Responsive to Nabiximols in a Patient with Multiple Sclerosis. Mult. Scler. Relat. Disord..

[15] Kalant H. (2004). Adverse Effects of Cannabis on Health: An Update of the Literature since 1996. Prog. Neuropsychopharmacol. Biol. Psychiatry.

[16] Lafaye G., Karila L., Blecha L., Benyamina A. (2017). Cannabis, Cannabinoids, and Health. Dialogues Clin. Neurosci..

[17] Hossain M.Z., Ando H., Unno S., Kitagawa J. (2020). Targeting Peripherally Restricted Cannabinoid Receptor 1, Cannabinoid Receptor 2, and Endocannabinoid-Degrading Enzymes for the Treatment of Neuropathic Pain Including Neuropathic Orofacial Pain. Int. J. Mol. Sci..

[18] Donvito G., Nass S.R., Wilkerson J.L., Curry Z.A., Schurman L.D., Kinsey S.G., Lichtman A.H. (2018). The Endogenous Cannabinoid System: A Budding Source of Targets for Treating Inflammatory and Neuropathic Pain. Neuropsychopharmacology.

[19] O’Hearn S., Diaz P., Wan B.A., DeAngelis C., Lao N., Malek L., Chow E., Blake A. (2017). Modulating the Endocannabinoid Pathway as Treatment for Peripheral Neuropathic Pain: A Selected Review of Preclinical Studies. Ann. Palliat. Med..

[20] Kamimura R., Hossain M.Z., Unno S., Ando H., Masuda Y., Takahashi K., Otake M., Saito I., Kitagawa J. (2018). Inhibition of 2-Arachydonoylgycerol Degradation Attenuates Orofacial Neuropathic Pain in Trigeminal Nerve-Injured Mice. J. Oral. Sci..

[21] Liang Y.-C., Huang C.-C., Hsu K.-S. (2007). The Synthetic Cannabinoids Attenuate Allodynia and Hyperalgesia in a Rat Model of Trigeminal Neuropathic Pain. Neuropharmacology.

[22] Clapper J.R., Moreno-Sanz G., Russo R., Guijarro A., Vacondio F., Duranti A., Tontini A., Sanchini S., Sciolino N.R., Spradley J.M. (2010). Anandamide Suppresses Pain Initiation through a Peripheral Endocannabinoid Mechanism. Nat. Neurosci..

[23] Moreno-Sanz G., Barrera B., Guijarro A., d’Elia I., Otero J.A., Alvarez A.I., Bandiera T., Merino G., Piomelli D. (2011). The ABC Membrane Transporter ABCG2 Prevents Access of FAAH Inhibitor URB937 to the Central Nervous System. Pharmacol. Res..

[24] Demartini C., Greco R., Zanaboni A.M., Francesconi O., Nativi C., Tassorelli C., Deseure K. (2018). Antagonism of Transient Receptor Potential Ankyrin Type-1 Channels as a Potential Target for the Treatment of Trigeminal Neuropathic Pain: Study in an Animal Model. Int. J. Mol. Sci..

[25] Deseure K., Hans G.H. (2015). Chronic Constriction Injury of the Rat’s Infraorbital Nerve (IoN-CCI) to Study Trigeminal Neuropathic Pain. J. Vis. Exp..

[26] Jhaveri M.D., Richardson D., Chapman V. (2007). Endocannabinoid Metabolism and Uptake: Novel Targets for Neuropathic and Inflammatory Pain. Br. J. Pharmacol..

[27] Jiang H., Ke B., Liu J., Ma G., Hai K., Gong D., Yang Z., Zhou C. (2019). Inhibition of Fatty Acid Amide Hydrolase Improves Depressive-Like Behaviors Independent of Its Peripheral Antinociceptive Effects in a Rat Model of Neuropathic Pain. Anesth. Analg..

[28] Sasso O., Bertorelli R., Bandiera T., Scarpelli R., Colombano G., Armirotti A., Moreno-Sanz G., Reggiani A., Piomelli D. (2012). Peripheral FAAH Inhibition Causes Profound Antinociception and Protects against Indomethacin-Induced Gastric Lesions. Pharmacol. Res..

[29] Guindon J., Lai Y., Takacs S.M., Bradshaw H.B., Hohmann A.G. (2013). Alterations in Endocannabinoid Tone Following Chemotherapy-Induced Peripheral Neuropathy: Effects of Endocannabinoid Deactivation Inhibitors Targeting Fatty-Acid Amide Hydrolase and Monoacylglycerol Lipase in Comparison to Reference Analgesics Following Cisplatin Treatment. Pharmacol. Res..

[30] Thompson J.M., Blanton H.L., Pietrzak A., Little W., Sherfey C., Guindon J. (2019). Front and Hind Paw Differential Analgesic Effects of Amitriptyline, Gabapentin, Ibuprofen, and URB937 on Mechanical and Cold Sensitivity in Cisplatin-Induced Neuropathy. Mol. Pain.

[31] Slivicki R.A., Saberi S.A., Iyer V., Vemuri V.K., Makriyannis A., Hohmann A.G. (2018). Brain-Permeant and -Impermeant Inhibitors of Fatty Acid Amide Hydrolase Synergize with the Opioid Analgesic Morphine to Suppress Chemotherapy-Induced Neuropathic Nociception Without Enhancing Effects of Morphine on Gastrointestinal Transit. J. Pharmacol. Exp. Ther..

[32] Sasso O., Wagner K., Morisseau C., Inceoglu B., Hammock B.D., Piomelli D. (2015). Peripheral FAAH and Soluble Epoxide Hydrolase Inhibitors Are Synergistically Antinociceptive. Pharmacol. Res..

[33] Greco R., Demartini C., Zanaboni A., Casini I., De Icco R., Reggiani A., Misto A., Piomelli D., Tassorelli C. (2021). Characterization of the Peripheral FAAH Inhibitor, URB937, in Animal Models of Acute and Chronic Migraine. Neurobiol. Dis..

[34] Greco R., Demartini C., Zanaboni A.M., Tumelero E., Reggiani A., Misto A., Piomelli D., Tassorelli C. (2020). FAAH Inhibition as a Preventive Treatment for Migraine: A Pre-Clinical Study. Neurobiol. Dis..

[35] Greco R., Francavilla M., Demartini C., Zanaboni A.M., Facchetti S., Palmisani M., Franco V., Tassorelli C. (2023). Activity of FAAH-Inhibitor JZP327A in an Experimental Rat Model of Migraine. Int. J. Mol. Sci..

[36] Greco R., Demartini C., Zanaboni A.M., Francavilla M., Reggiani A., Realini N., Scarpelli R., Piomelli D., Tassorelli C. (2022). Potentiation of Endocannabinoids and Other Lipid Amides Prevents Hyperalgesia and Inflammation in a Pre-Clinical Model of Migraine. J. Headache Pain.

[37] Wen J., Sackett S., Tanaka M., Zhang Y. (2023). Therapeutic Effects of Combined Treatment with the AEA Hydrolysis Inhibitor PF04457845 and the Substrate Selective COX-2 Inhibitor LM4131 in the Mouse Model of Neuropathic Pain. Cells.

[38] Tanaka M., Yagyu K., Sackett S., Zhang Y. (2019). Anti-Inflammatory Effects by Pharmacological Inhibition or Knockdown of Fatty Acid Amide Hydrolase in BV2 Microglial Cells. Cells.

[39] Rock E.M., Moreno-Sanz G., Limebeer C.L., Petrie G.N., Angelini R., Piomelli D., Parker L.A. (2017). Suppression of Acute and Anticipatory Nausea by Peripherally Restricted Fatty Acid Amide Hydrolase Inhibitor in Animal Models: Role of PPARα and CB _1_ Receptors. Br. J. Pharmacol..

[40] Echeverry S., Shi X.Q., Rivest S., Zhang J. (2011). Peripheral Nerve Injury Alters Blood-Spinal Cord Barrier Functional and Molecular Integrity through a Selective Inflammatory Pathway. J. Neurosci..

[41] Fried N.T., Maxwell C.R., Elliott M.B., Oshinsky M.L. (2018). Region-Specific Disruption of the Blood-Brain Barrier Following Repeated Inflammatory Dural Stimulation in a Rat Model of Chronic Trigeminal Allodynia. Cephalalgia.

[42] DosSantos M.F., Holanda-Afonso R.C., Lima R.L., DaSilva A.F., Moura-Neto V. (2014). The Role of the Blood Brain Barrier in the Development and Treatment of Migraine and Other Pain Disorders. Front. Cell Neurosci..

[43] Mitrirattanakul S., Ramakul N., Guerrero A.V., Matsuka Y., Ono T., Iwase H., Mackie K., Faull K.F., Spigelman I. (2006). Site-Specific Increases in Peripheral Cannabinoid Receptors and Their Endogenous Ligands in a Model of Neuropathic Pain. Pain.

[44] Lim G., Sung B., Ji R.-R., Mao J. (2003). Upregulation of Spinal Cannabinoid-1-Receptors Following Nerve Injury Enhances the Effects of Win 55,212-2 on Neuropathic Pain Behaviors in Rats. Pain.

[45] Petrosino S., Palazzo E., de Novellis V., Bisogno T., Rossi F., Maione S., Di Marzo V. (2007). Changes in Spinal and Supraspinal Endocannabinoid Levels in Neuropathic Rats. Neuropharmacology.

[46] Starowicz K., Makuch W., Korostynski M., Malek N., Slezak M., Zychowska M., Petrosino S., De Petrocellis L., Cristino L., Przewlocka B. (2013). Full Inhibition of Spinal FAAH Leads to TRPV1-Mediated Analgesic Effects in Neuropathic Rats and Possible Lipoxygenase-Mediated Remodeling of Anandamide Metabolism. PLoS ONE.

[47] Barrie N., Manolios N. (2017). The Endocannabinoid System in Pain and Inflammation: Its Relevance to Rheumatic Disease. Eur. J. Rheumatol..

[48] Latremoliere A., Woolf C.J. (2009). Central Sensitization: A Generator of Pain Hypersensitivity by Central Neural Plasticity. J. Pain.

[49] Price T.J., Helesic G., Parghi D., Hargreaves K.M., Flores C.M. (2003). The Neuronal Distribution of Cannabinoid Receptor Type 1 in the Trigeminal Ganglion of the Rat. Neuroscience.

[50] Tsou K., Brown S., Sañudo-Peña M.C., Mackie K., Walker J.M. (1998). Immunohistochemical Distribution of Cannabinoid CB1 Receptors in the Rat Central Nervous System. Neuroscience.

[51] Ellis A., Bennett D.L.H. (2013). Neuroinflammation and the Generation of Neuropathic Pain. Br. J. Anaesth..

[52] Zhuang Z.-Y., Wen Y.-R., Zhang D.-R., Borsello T., Bonny C., Strichartz G.R., Decosterd I., Ji R.-R. (2006). A Peptide C-Jun N-Terminal Kinase (JNK) Inhibitor Blocks Mechanical Allodynia after Spinal Nerve Ligation: Respective Roles of JNK Activation in Primary Sensory Neurons and Spinal Astrocytes for Neuropathic Pain Development and Maintenance. J. Neurosci..

[53] Costa B., Comelli F., Bettoni I., Colleoni M., Giagnoni G. (2008). The Endogenous Fatty Acid Amide, Palmitoylethanolamide, Has Anti-Allodynic and Anti-Hyperalgesic Effects in a Murine Model of Neuropathic Pain: Involvement of CB(1), TRPV1 and PPARgamma Receptors and Neurotrophic Factors. Pain.

[54] Lo Verme J., Fu J., Astarita G., La Rana G., Russo R., Calignano A., Piomelli D. (2005). The Nuclear Receptor Peroxisome Proliferator-Activated Receptor-Alpha Mediates the Anti-Inflammatory Actions of Palmitoylethanolamide. Mol. Pharmacol..

[55] Seol T.-K., Lee W., Park S., Kim K.N., Kim T.Y., Oh Y.N., Jun J.H. (2017). Effect of Palmitoylethanolamide on Inflammatory and Neuropathic Pain in Rats. Korean J. Anesth..

[56] Kamper D. (2022). Palmitoylethanolamide (PEA) in the Treatment of Neuropathic Pain: A Case Study. Nutr. Health.

[57] Lang-Illievich K., Klivinyi C., Lasser C., Brenna C.T.A., Szilagyi I.S., Bornemann-Cimenti H. (2023). Palmitoylethanolamide in the Treatment of Chronic Pain: A Systematic Review and Meta-Analysis of Double-Blind Randomized Controlled Trials. Nutrients.

[58] Muccioli G.G., Stella N. (2008). Microglia Produce and Hydrolyze Palmitoylethanolamide. Neuropharmacology.

[59] Núñez E., Benito C., Tolón R.M., Hillard C.J., Griffin W.S.T., Romero J. (2008). Glial Expression of Cannabinoid CB2 Receptors and Fatty Acid Amide Hydrolase Are Beta Amyloid–Linked Events in Down’s Syndrome. Neuroscience.

[60] Benito C., Núñez E., Tolón R.M., Carrier E.J., Rábano A., Hillard C.J., Romero J. (2003). Cannabinoid CB2 Receptors and Fatty Acid Amide Hydrolase Are Selectively Overexpressed in Neuritic Plaque-Associated Glia in Alzheimer’s Disease Brains. J. Neurosci..

[61] Grieco M., De Caris M.G., Maggi E., Armeli F., Coccurello R., Bisogno T., D’Erme M., Maccarrone M., Mancini P., Businaro R. (2021). Fatty Acid Amide Hydrolase (FAAH) Inhibition Modulates Amyloid-Beta-Induced Microglia Polarization. Int. J. Mol. Sci..

[62] Takeda M., Matsumoto S., Sessle B.J., Shinoda M., Iwata K. (2011). Peripheral and Central Mechanisms of Trigeminal Neuropathic and Inflammatory Pain. J. Oral Biosci..

[63] Bista P., Imlach W.L. (2019). Pathological Mechanisms and Therapeutic Targets for Trigeminal Neuropathic Pain. Medicines.

[64] Shinoda M., Imamura Y., Hayashi Y., Noma N., Okada-Ogawa A., Hitomi S., Iwata K. (2021). Orofacial Neuropathic Pain-Basic Research and Their Clinical Relevancies. Front. Mol. Neurosci..

[65] Wang W., Wang W., Mei X., Huang J., Wei Y., Wang Y., Wu S., Li Y. (2009). Crosstalk between Spinal Astrocytes and Neurons in Nerve Injury-Induced Neuropathic Pain. PLoS ONE.

[66] Hossain M., Unno S., Ando H., Masuda Y., Kitagawa J. (2017). Neuron–Glia Crosstalk and Neuropathic Pain: Involvement in the Modulation of Motor Activity in the Orofacial Region. Int. J. Mol. Sci..

[67] Zhang T., Zhang M., Cui S., Liang W., Jia Z., Guo F., Ou W., Wu Y., Zhang S. (2023). The Core of Maintaining Neuropathic Pain: Crosstalk between Glial Cells and Neurons (Neural Cell Crosstalk at Spinal Cord). Brain Behav..

[68] Matejuk A., Ransohoff R.M. (2020). Crosstalk Between Astrocytes and Microglia: An Overview. Front. Immunol..

[69] Qi J., Chen C., Meng Q.-X., Wu Y., Wu H., Zhao T.-B. (2016). Crosstalk between Activated Microglia and Neurons in the Spinal Dorsal Horn Contributes to Stress-Induced Hyperalgesia. Sci. Rep..

[70] Guo W., Wang H., Watanabe M., Shimizu K., Zou S., LaGraize S.C., Wei F., Dubner R., Ren K. (2007). Glial–Cytokine–Neuronal Interactions Underlying the Mechanisms of Persistent Pain. J. Neurosci..

[71] Stella N. (2010). Cannabinoid and Cannabinoid-like Receptors in Microglia, Astrocytes, and Astrocytomas. Glia.

[72] Meza R.C., Ancatén-González C., Chiu C.Q., Chávez A.E. (2022). Transient Receptor Potential Vanilloid 1 Function at Central Synapses in Health and Disease. Front. Cell Neurosci..

[73] Chistyakov D.V., Aleshin S.E., Astakhova A.A., Sergeeva M.G., Reiser G. (2015). Regulation of Peroxisome Proliferator-Activated Receptors (PPAR) α and -γ of Rat Brain Astrocytes in the Course of Activation by Toll-like Receptor Agonists. J. Neurochem..

[74] Keppel Hesselink J.M., Kopsky D.J., Witkamp R.F. (2014). Palmitoylethanolamide (PEA)—‘Promiscuous’ Anti-Inflammatory and Analgesic Molecule at the Interface between Nutrition and Pharma. PharmaNutrition.

[75] Nagarkatti P., Pandey R., Rieder S.A., Hegde V.L., Nagarkatti M. (2009). Cannabinoids as Novel Anti-Inflammatory Drugs. Future Med. Chem..

[76] Sancho R., Calzado M.A., Di Marzo V., Appendino G., Muñoz E. (2003). Anandamide Inhibits Nuclear Factor-KappaB Activation through a Cannabinoid Receptor-Independent Pathway. Mol. Pharmacol..

[77] Klein T.W., Cabral G.A. (2006). Cannabinoid-Induced Immune Suppression and Modulation of Antigen-Presenting Cells. J. Neuroimmune Pharmacol..

[78] Costa B., Siniscalco D., Trovato A.E., Comelli F., Sotgiu M.L., Colleoni M., Maione S., Rossi F., Giagnoni G. (2006). AM404, an Inhibitor of Anandamide Uptake, Prevents Pain Behaviour and Modulates Cytokine and Apoptotic Pathways in a Rat Model of Neuropathic Pain. Br. J. Pharmacol..

[79] Paszcuk A.F., Dutra R.C., da Silva K.A.B.S., Quintão N.L.M., Campos M.M., Calixto J.B. (2011). Cannabinoid Agonists Inhibit Neuropathic Pain Induced by Brachial Plexus Avulsion in Mice by Affecting Glial Cells and MAP Kinases. PLoS ONE.

[80] Justinova Z., Mangieri R.A., Bortolato M., Chefer S.I., Mukhin A.G., Clapper J.R., King A.R., Redhi G.H., Yasar S., Piomelli D. (2008). Fatty Acid Amide Hydrolase Inhibition Heightens Anandamide Signaling without Producing Reinforcing Effects in Primates. Biol. Psychiatry.

[81] Gobbi G., Bambico F.R., Mangieri R., Bortolato M., Campolongo P., Solinas M., Cassano T., Morgese M.G., Debonnel G., Duranti A. (2005). Antidepressant-like Activity and Modulation of Brain Monoaminergic Transmission by Blockade of Anandamide Hydrolysis. Proc. Natl. Acad. Sci. USA.

[82] Jayamanne A., Greenwood R., Mitchell V.A., Aslan S., Piomelli D., Vaughan C.W. (2006). Actions of the FAAH Inhibitor URB597 in Neuropathic and Inflammatory Chronic Pain Models. Br. J. Pharmacol..

[83] Hama A.T., Germano P., Varghese M.S., Cravatt B.F., Milne G.T., Pearson J.P., Sagen J. (2014). Fatty Acid Amide Hydrolase (FAAH) Inhibitors Exert Pharmacological Effects, but Lack Antinociceptive Efficacy in Rats with Neuropathic Spinal Cord Injury Pain. PLoS ONE.

[84] Vozella V., Ahmed F., Choobchian P., Merrill C.B., Zibardi C., Tarzia G., Mor M., Duranti A., Tontini A., Rivara S. (2019). Pharmacokinetics, Pharmacodynamics and Safety Studies on URB937, a Peripherally Restricted Fatty Acid Amide Hydrolase Inhibitor, in Rats. J. Pharm. Pharmacol..

[85] Hurley R.W., Adams M.C.B. (2008). Sex, Gender, and Pain: An Overview of a Complex Field. Anesth. Analg..

[86] Vos B.P., Strassman A.M., Maciewicz R.J. (1994). Behavioral Evidence of Trigeminal Neuropathic Pain Following Chronic Constriction Injury to the Rat’s Infraorbital Nerve. J. Neurosci..

[87] Deseure K., Koek W., Colpaert F.C., Adriaensen H. (2002). The 5-HT1A Receptor Agonist F 13640 Attenuates Mechanical Allodynia in a Rat Model of Trigeminal Neuropathic Pain. Eur. J. Pharmacol..

[88] Waite P.M.E., Ashwell K.W.S. (2004). Trigeminal Sensory System. The Human Nervous System.

[89] Terayama R., Nagamatsu N., Ikeda T., Nakamura T., Rahman O.I.F., Sakoda S., Shiba R., Nishimori T. (1997). Differential Expression of Fos Protein after Transection of the Rat Infraorbital Nerve in the Trigeminal Nucleus Caudalis. Brain Res..

[90] Panneton W.M., Pan B., Gan Q. (2017). Somatotopy in the Medullary Dorsal Horn as a Basis for Orofacial Reflex Behavior. Front. Neurol..

[91] Demartini C., Greco R., Magni G., Zanaboni A.M., Riboldi B., Francavilla M., Nativi C., Ceruti S., Tassorelli C. (2022). Modulation of Glia Activation by TRPA1 Antagonism in Preclinical Models of Migraine. Int. J. Mol. Sci..

