# Supplementary material for: URB937 Prevents the Development of Mechanical Allodynia in Male Rats with Trigeminal Neuralgia
Source: Pharmaceuticals (Basel). 2023 Nov 18;16(11):1626. doi: 10.3390/ph16111626 (PMC10675761; doi:10.3390/ph16111626)

Figure S1. Open field test.

On day +25, after MST, rats were treated with URB937 or vehicle daily for 5 days. On day +29, 1 hour after URB937 or vehicle treatment, animals were placed in the open-field arena (92 × 92 cm) and recorded for 10 min. The distance (expressed in meters) travelled in the apparatus (A), the average speed (expressed in meters/seconds) (B) and the total time immobile (expressed in seconds) (C) were analyzed by means of the ANY-Maze software (Ugo Basile, application version 4.99 g Beta). (D) Track plots of the different experimental groups. Data were tested with Kruskal-Wallis test followed by Dunn's multiple comparisons test since data were not normally distributed (Kolmogorov-Smirnov normality test). Data are expressed as mean ± SEM; N = 4

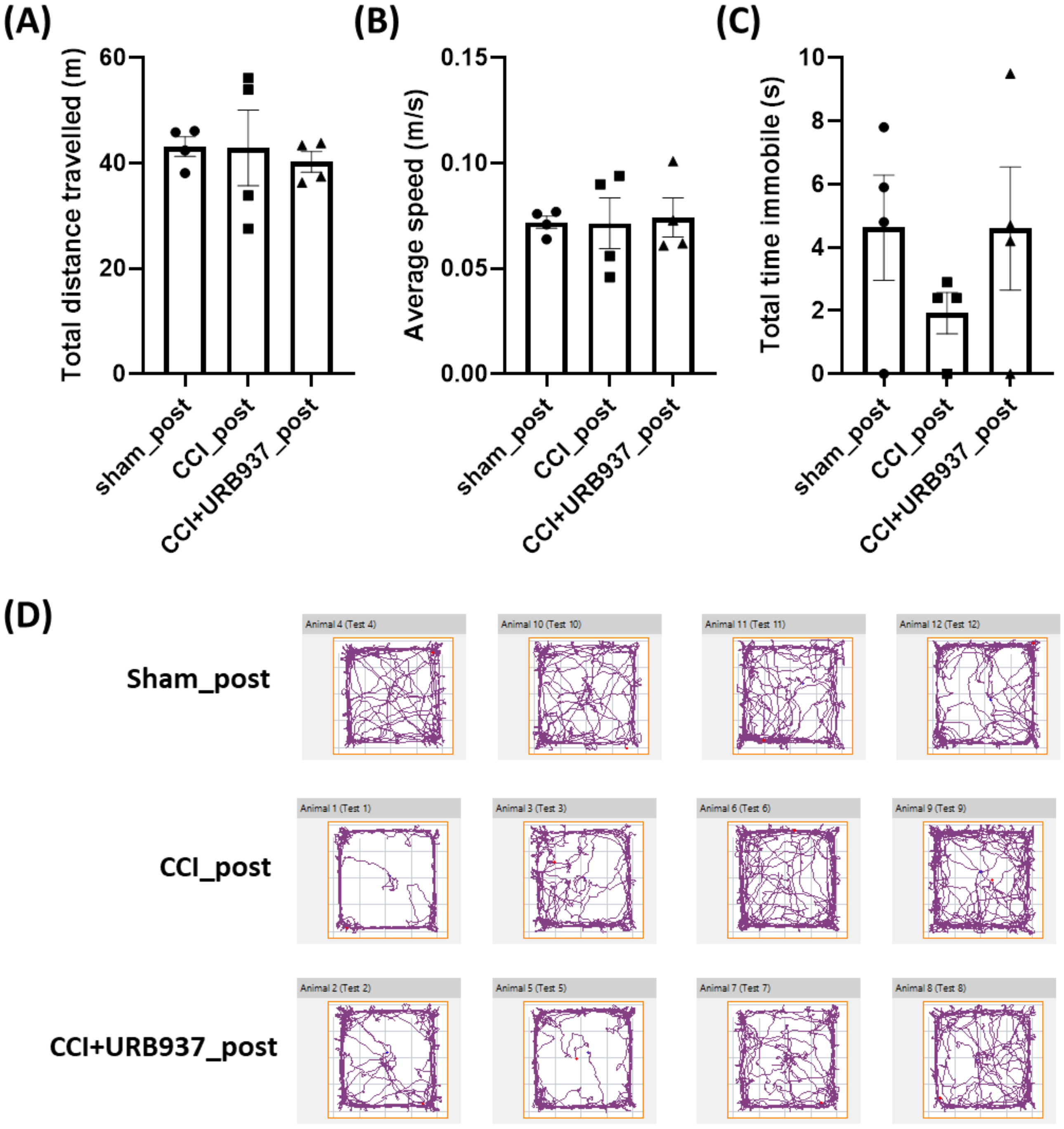

Supplement: Supplementary file 1 [file pharmaceuticals-16-01626-s001.zip › pharmaceuticals-2606797-supplementary.pdf]
